# Changing Time Horizons and Trust: Experiences of Aging With Hemophilia

**DOI:** 10.1093/geroni/igab046.022

**Published:** 2021-12-17

**Authors:** Tam Perry, Sara Schwartz

**Affiliations:** 1 Wayne State University, Detroit, Michigan, United States; 2 University of Southern California, Los Angeles, California, United States

## Abstract

Trust among those who have experienced a lifetime of medical encounters warrants attention to how trust is both cumulative and complex. This study of a historically isolated cohort incorporates interviews (n=25 older adults/professionals) and focus groups uses a lens of trust to highlight the experiences of those aging with hemophilia, individuals who never expected to age. Understood through the lens of trust, the data show evidence of the absence of safe spaces particularly during the early 80s - blood contamination concerns and homophobia-leading often to social withdrawal. Over time, however, some individuals and families created trusted venues to begin demanding research, treatment and policy change. Advocacy re-engaged the community to organize, educate and advance safety protocols for blood product manufacturing and distribution. This presentation will illuminate how experiences with medical providers, contaminated blood supplies, stigma and uncertain in other spheres of one’s life make trust a co-constructed, fragile concept.

